# The multifaceted role of gibberellins in somatic embryogenesis: from *in vitro* culture to molecular networks

**DOI:** 10.3389/fpls.2026.1868680

**Published:** 2026-07-27

**Authors:** Maja Belić, Snežana Zdravković-Korać, Jelena Milojević

**Affiliations:** Department of Plant Physiology, Institute for Biological Research “Siniša Stanković” - National Institute of the Republic of Serbia, University of Belgrade, Belgrade, Serbia

**Keywords:** endogenous gibberellins, gene expression, gibberellins, somatic embryogenesis, tissue culture

## Abstract

Gibberellins (GAs) are central regulators of plant growth and development, yet their involvement in somatic embryogenesis (SE) has received limited attention and often produces contradictory results. This review critically examines the current knowledge on the role of GAs in SE by integrating evidence from physiological, molecular, and developmental studies across a wide range of plant species. Available data indicate that exogenous GAs and GA biosynthesis inhibitors can either promote or inhibit the induction, progression, and conversion of somatic embryos, depending on the species, genotype, explant origin, developmental stage, and culture conditions. These opposing effects underscore that exogenous responses are tightly linked to endogenous GA homeostasis. Particular attention is paid to GA metabolism and signaling genes, whose expression patterns during SE reveal substantial interspecific and genotypic variation. Endogenous GA profiles reveal that both high and low levels of bioactive GAs correlate with SE induction across different systems, suggesting that endogenous GA levels may be a key determinant of embryogenic competence and may underlie major differences in regeneration capacity among species and genotypes. Furthermore, GAs function within a broader regulatory network through extensive crosstalk with other hormones (auxins, abscisic acid, cytokinins, and ethylene), environmental factors (e.g., light and temperature) and interactions with major embryogenesis-related transcription factors such as LEC1, LEC2, FUS3, ABI3, AGL15, AGL18, and BBM. These interactions position GAs as dynamic components of a multilayered hormonal and transcriptional framework that controls the switch from somatic to embryogenic development. Integrative approaches—including hormone profiling, gene expression analysis, functional genetics, and developmental studies—are essential to elucidate the precise function of GAs in SE and thereby improve protocols for plant regeneration.

## 1 Introduction

Somatic embryogenesis (SE) is a process in which somatic cells transition from a vegetative to an embryonic developmental pathway without gamete formation or fertilization (von Arnold et al., 2002; Jha et al., 2020). Owing to their totipotency, plant somatic cells can undergo redifferentiation followed by cell division, eventually leading to the redifferentiation of a subset of cells into somatic embryos that are capable of developing into complete plants. This plant-specific phenomenon was first reported in the 1950s in carrot cell suspension cultures (Reinert, 1958; Steward et al., 1958). Even at that time, it was recognized that *in vitro* SE offered substantial potential for both fundamental research and applied plant biotechnology.

During SE, competent somatic cells undergo cellular reprogramming and proliferate to form multicellular structures that progress through well-defined morphological stages. In dicotyledonous species, these stages typically include the globular, heart, torpedo, and cotyledonary stages, whereas in monocotyledonous species embryogenesis generally proceeds through the globular, scutellar, and coleoptilar stages, closely resembling the corresponding stages of zygotic embryogenesis. This enables direct, continuous observation under controlled *in vitro* conditions, making SE a powerful model system for studying the regulation of embryogenesis. SE is often considered a particularly advantageous regeneration system under *in vitro* conditions because complete plants can be regenerated through a single developmental process rather than through successive shoot and root formation, as occurs during *de novo* organogenesis. Moreover, the ability to maintain embryogenic cultures for extended periods has enabled its widespread use in large-scale clonal propagation and artificial seed technology (Meucci et al., 2024; Martínez and Corredoira, 2024). Despite these advantages, many species and genotypes remain recalcitrant to SE, underscoring the need for a deeper understanding of the underlying molecular, epigenetic, and physiological mechanisms.

The expression of embryogenic competence depends on the physiological state of the cell, which is determined by a complex interplay of genetic and exogenous factors (von Arnold et al., 2002; Fehér, 2008). These factors activate complex signaling networks, resulting in altered gene expression and changes in endogenous hormone levels (Jiménez, 2005). In addition to stress-related factors such as heat, chilling, drought, osmotic, and heavy metal stress, plant growth regulators (PGRs) are among the most important determinants of SE induction (Nic-Can et al., 2016). The roles of auxins and cytokinins in SE induction have been extensively studied, as they are required for SE induction in most plant species (von Arnold et al., 2002; Jiménez, 2005; Salaün et al., 2021; Elhiti and Stasolla, 2022; Asghar et al., 2023; Kumar et al., 2025). However, considerably less is known about the roles of other PGRs, particularly gibberellins (GAs), in SE.

GAs are involved in nearly all aspects of plant development and form key components of the complex hormonal regulatory network (Shani et al., 2024). They play essential roles in processes such as seed germination, pollen maturation, trichome development, leaf expansion, and flowering induction (Sauter et al., 1995; Sakamoto et al., 2004; Anderson et al., 2001; Plackett et al., 2012; Hedden and Sponsel, 2015; Hedden, 2016; Plackett and Wilson, 2016). However, the role of GAs in SE is complex and often contradictory. In most plant species, GAs inhibit SE induction and somatic embryo development (Chen and Chang, 2003; Wang et al., 2004; George et al., 2008; Subotić et al., 2009; Moradi et al., 2017), whereas in certain species they are essential for successful SE induction (Komai et al., 1996a, b; Ruduś et al., 2001; Thomas, 2006; Belić et al., 2020b).

Most studies on GA function in SE have relied on the exogenous application of GA_3_ or GA biosynthesis inhibitors to plant organs and tissues at various developmental stages. Although these approaches have provided valuable insights, the molecular mechanisms of GA action, its interactions with other regulatory factors, and the dynamics of endogenous GA levels during SE remain poorly understood. Consequently, the involvement of GAs in SE has received only limited attention in previous reviews (Jiménez, 2005; Yang and Zhang, 2010; Elhiti et al., 2013; Kumar and van Staden, 2017; Kępczyńska and Kępczyński, 2023; Rose, 2019). To our knowledge, no review has been devoted specifically to the impact of GAs on SE induction. This review therefore aims to summarize and critically assess current knowledge of the mechanisms underlying GA action in SE.

## 2 GA biosynthesis and signal transduction

**Figure 1:**
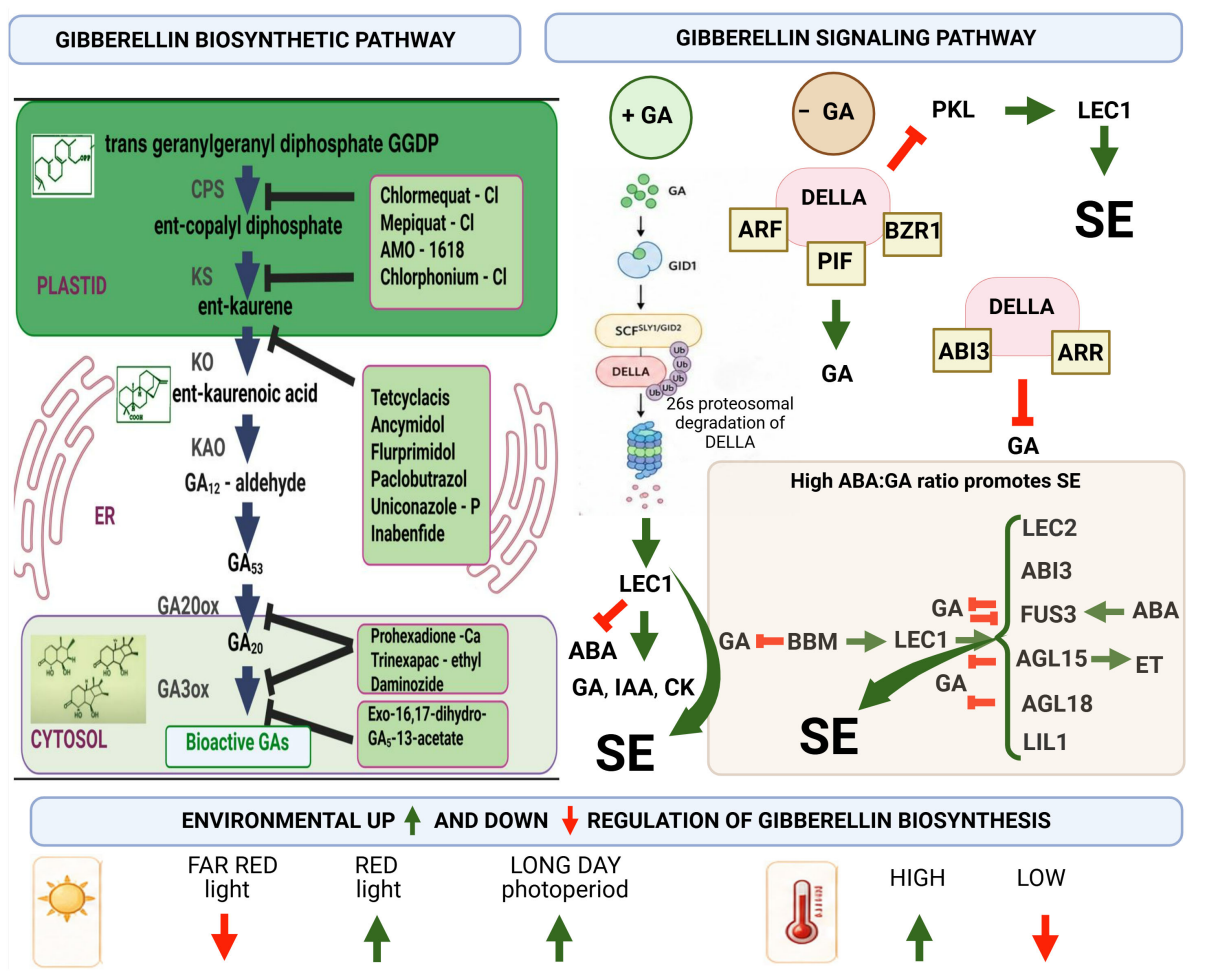
Integrative overview of gibberellin biosynthesis, signaling, hormonal crosstalk, and environmental regulation during somatic embryogenesis. CPS, ent-copalyl diphosphate synthase; KS, ent-kaurene synthase; KO, ent-kaurene oxidase; KAO, ent-kaurenoic acid oxidase; GA20ox, gibberellin 20-oxidase; GA3ox, gibberellin 3-oxidase; GAs, gibberellins; TF, transcription factor; GID1, GIBBERELLIN INSENSITIVE DWARF1 receptor; Ub, ubiquitin; DELLA, DELLA repressor protein; ARF, Auxin Response Factor; PIF, Phytochrome Interacting Factor; BZR, BRASSINAZOLE-RESISTANT transcription factor; ABI3, ABA INSENSITIVE 3 transcription factor; LEC1, LEAFY COTYLEDON1, LEC2, LEAFY COTYLEDON2; BBM, BABY BOOM; FUS3, FUSCA3; AGL15,18, AGAMOUS-LIKE15,18; LIL1, LEAFY COTYLEDON LIKE1; PKL, PICKLE; ARR, Arabidopsis Response Regulator; GA, gibberellic acid; ABA, Abscisic acid; IAA, Indole-3-acetic acid; CK, Cytokinin; ET, Ethylene. Arrows indicate activation or promotion, whereas blunt-ended lines indicate repression or inhibition. The graph was created in BioRender (Agreement number: CC29VPUIWZ) https://BioRender.com/wp6sntz; last accessed (25.06.2026.).

GAs constitute a large family of diterpenoid compounds, of which only a few—exhibit biological activity. Among these, GA_1_ and GA_4_ are the principal endogenous bioactive GAs in most plant species, whereas GA_3_ and GA_7_ occur and function as bioactive GAs in a more limited range of species (Yamaguchi, 2008; Hedden, 2016, 2020). Their biosynthesis occurs in three phases across different cellular compartments. In plastids, geranylgeranyl diphosphate (GGPP) is converted to ent-kaurene by ent-copalyl diphosphate synthase and ent-kaurene synthase. In the endoplasmic reticulum, ent-kaurene is oxidized to GA_12_ by ent-kaurene oxidase and ent-kaurenoic acid oxidase. The final steps of GA biosynthesis occur in the cytosol, where GA20-oxidases (GA20ox) catalyze successive C-20 oxidation reactions leading to the formation of GA precursors, while GA3-oxidases (GA3ox) catalyze the final 3β-hydroxylation step that produces bioactive GAs. In contrast, GA2-oxidases (GA2ox) inactivate them by 2β-hydroxylation (e.g., GA_1_ → GA_8_, GA_4_ → GA_34_) (Coles et al., 1999; Olszewski et al., 2002; Hedden and Thomas, 2012). These later enzymes are encoded by multigene families with tissue- and stage-specific expression patterns (Sakamoto et al., 2004; Rieu et al., 2008; Han and Zhu, 2011) (**Figure 1**).

Bioactive GAs initiate signaling by binding to the GIBBERELLIN INSENSITIVE DWARF1 (GID1), receptor, thereby promoting its interaction with DELLA proteins, the main repressors of GA responses (Ueguchi-Tanaka et al., 2005). The resulting GA–GID1–DELLA complex recruits the F-box protein SLEEPY1, leading to polyubiquitination of DELLA proteins by the SCF^SLY1/GID2^ E3 ligase and their subsequent degradation by the 26S proteasome (Lechner et al., 2006). This relieves DELLA-mediated repression of GA-responsive genes. In the absence of GA, DELLA proteins remain stable and actively suppress GA signaling (Dill et al., 2004; Gao et al., 2011; Wallner et al., 2016). In addition, DELLA proteins act as master growth regulators that interact with numerous other regulators across cellular pathways in response to internal and external cues (**Figure 1**) (Shani et al., 2024).

To ensure proper progression of these physiological processes, plants have evolved tightly regulated mechanisms to control GA levels, including feedback regulation in which bioactive GAs modulate their own biosynthesis. Components of the GA signaling pathway, including the GID1 receptor, DELLA proteins, the F-box protein SLEEPY1, and transcription factors such as REPRESSION OF SHOOT GROWTH, YABBY, and AT-hook proteins, mediate this feedback by repressing *GA20ox* and *GA3ox* gene expression (**Figure 1**) (Ishida et al., 2004; Matsushita et al., 2007; Ueguchi-Tanaka et al., 2007; Zentella et al., 2007; Fukazawa et al., 2014; Boccaccini et al., 2014; Yang et al., 2016). GA20ox is particularly sensitive to this regulation and is often rate-limiting for bioactive GA production (Israelsson et al., 2004).

## 3 The role of exogenous GA and GA biosynthesis inhibitors in somatic embryogenesis

GAs have seldom been used for SE induction; therefore, their role in this process has been studied far less extensively than that of auxins and cytokinins. Among bioactive GAs, GA_3_ has been used almost exclusively for this purpose, typically in combination with auxin and/or cytokinin for SE induction or after PGR treatment to promote somatic embryo regeneration, germination, and conversion to plantlets (**Table 1**). Other bioactive GAs, such as GA_4_, GA_7_, and GA_1_, have been applied far less frequently in agriculture (Rademacher, 2015) and almost never for SE induction. The notable exception is SE induction in spinach, where both GA_3_ and GA_1_ were successfully used, although GA_3_ proved more effective than GA_1_ (Belić et al., 2020b), most likely because of its greater persistence in plant tissues (Rademacher, 2015).

**Table 1:** Effects of exogenous GAs on the induction of somatic embryos across various explant types and species.

Plant species	Cultivar	Explant type	Callus inductions	Somatic embryo regeneration	References
*Hardwicka binata*	/	cotyledons	MS + 4.64 µM Kin + 5.37 µM NAA	MS + 2052.6 µM + L-glutamine + 0.084 µM GA_3_	Das et al., 1995
*Cicer arietinum*	Macrocarpa	cotyledons	B5 + 13.7 µM Zeatin + 0.2 µM IAA + 1.5 % sucrose	½ B5 + 14. 4 µM GA_3_ + 1 µM IBA	Hita et al., 1997
*Rosa hybrida*	Carefree Beauty Baby Katie	leaves	½ MS + 100 µM 2.4-D + 59 mM sucrose + 3 µM tiamin	½ MS + 23 µM TDZ + 3 µM GA_3_ + 3 μM tiamin + 110 nm glucose	Hsia and Korban, 1996
	Carefree Beauty	Leaves and petiole	½ MS + 2.9 µM GA_3_ + 2.3 TDZ or 2.9 GA_3_ + 2.2 µM BA	1/2MS + 3.8 µM ABA	Li et al., 2002
	Landora	leaves	1/2MS + 2.2 µM BA + 5.4 µM NAA + 2.2 µM 2,4-D + 3% sucrose	½ MS + 2.2 µM BA + 0.05 µM NAA + 0.3 µM GA_3_ + 600 mg/l prolin	Rout et al., 1991
		steams	1/2MS + 2.2 µM BA + 5.4 µM NAA + 9 µM 2,4-D + 3% sucrose		
*Zea mays*	Gaurav	zygotic embryos	MS + 5 mg/l 2.4 -D + 2 mg/l NAA + 1 mg/l BAP + 3 % sucrose	MS + 50-150 µM GA_3_ + 150 µM putrescin	Joshi et al., 2010
*Medicago sativa*	Rangelander	petiole	SH + 5 mg/l 2.4-D + 1 mg/l Kin + 5 µM GA_3_	SH + 5 µM GA_3_	Ruduś et al., 2001
*Foeniculum vulgare*	Miller	petiole	MS + 2 µM 2,4-D + 0.46 µM Kin + 2.9 µM GA_3_ + 4% sucrose	MS + 2.9 µM GA_3_ + 3% sucrose	Hunault and Maatar, 1995
*Iris germanica*	G1, Adorn, Rococo	leaves	MS + 1 mg/l 2.4-D +1 mg/l Kin + 250 mg/l prolin + 3% sucrose	MS + 1mg/l GA_3_ + 3 % sucrose	Shimizu et al., 1997
*Tylophora indica*	Merrill	Inter-nodal segments	MS + 4 µM 2.4-D	MS + 10 µmol GA_3_ + 200 mM sucrose	Thomas, 2006
*Spinacia oleracea*	Carpo	Hypocotyl segments	MS + 85.7 μM IAA + 100 μM GA_3_ + 2% sucrose	MS + 11.4 μM IAA + 10 μM GA_3_ + 2% sucrose	Xiao and Branchard, 1993
	Jiromaru, Nihon, Hoyo, Minsterland, Viroflay, King of Denmark, Nobel, Ujo	Cotyledon, hypocotyl, root segments, leaf fragments	MS/2 + 10 μM NAA + 0.1 μM GA_3_ + 1% sucrose	MS, PGR-free + 2% sucrose	Komai et al., 1996a
	Jiromaru	Root segments	MS/2 + 30 μM NAA + 100 μM GA_3_ + 1% sucrose	MS, PGR-free + 2% sucrose	Komai et al., 1996b
			MS/2 + 30 μM NAA + 100 μM GA_3_ + 29 mM fructose	MS, PGR-free + 59 mM sucrose	Komai et al., 1996c
	Nippon	Root segments	1/2 MS + 10 μM NAA + 0.1 μM GA_3_ + 5.4 g/l fructose	1/2 MS, PGR-free + 2% sucrose	Ishizaki et al., 2001
	Matador	Root segments	MS + 20 μM NAA + 5 μM GA_3_ + 2% sucrose	MS, 5 μM Kin + 2% sucrose	Milojević et al., 2011

The composition of plant growth regulators in the media used for embryogenic callus induction and somatic embryo regeneration is presented in the table.

The role of GAs in SE differs markedly among plant species, with both promotive and suppressive effects reported. In many plant species, exogenous GA_3_ inhibits SE induction, as shown in *Centaurium erythraea* (Subotić et al., 2009), *Oncidium* (Chen and Chang, 2003), *Phoenix dactylifera* (Hussam et al., 2015), *Pelargonium hortorum* (Hutchinson et al., 1997), and *Medicago truncatula* (Igielski and Kępczyńska, 2017). In several of these species, treatment with GA biosynthesis inhibitors such as ancymidol, uniconazole, and paclobutrazol (PAC) was associated with enhanced SE induction (Hutchinson et al., 1997; Chen and Chang, 2003; Subotić et al., 2009; Hussam et al., 2015).

In contrast, GA_3_ enhanced SE efficiency in *Foeniculum vulgare* (Hunault and Maatar, 1995), increased somatic embryo regeneration in *Rosa* spp (Li and Qu, 2002), and promoted both induction and differentiation of somatic embryos in *Medicago sativa* (Ruduś et al., 2001). Interestingly, exogenous GA_3_ exerted opposite effects in two species of the same genus: it enhanced SE induction in *Gentiana tibetica* but suppressed it in *G. cruciata* (Mikula et al., 1996). One of the most notable exceptions is spinach, in which GA_3_ is indispensable for efficient SE induction (Xiao and Branchard, 1993; Komai et al., 1996a, b; Ishizaki et al., 2001; Belić et al., 2020b). Remarkably, PAC acted synergistically with GA_3_, further enhancing SE efficiency in spinach root explants (Belić et al., 2022a). This observation suggests that the effects of PAC cannot be explained solely by reduced endogenous GA levels and that additional regulatory mechanisms are likely involved.

Although PAC is widely used as a GA biosynthesis inhibitor, increasing evidence indicates that its physiological effects extend beyond GA metabolism. PAC and related triazoles can influence multiple hormonal pathways by altering both hormone metabolism and signaling. In addition to suppressing GA biosynthesis by blocking the conversion of ent-kaurene to ent-kaurenoic acid, these compounds reduce the expression of genes involved in auxin, brassinosteroid, jasmonate, and ethylene biosynthesis and signaling, while enhancing the expression of genes associated with cytokinin and ABA metabolism and signaling (Seesangboon et al., 2018; Gazara et al., 2019; Duan et al., 2019; Desta and Amare, 2021; Castro-Camba et al., 2025). In addition, these inhibitors enhance the activity of antioxidant enzymes, thereby increasing stress tolerance (Nagar et al., 2021; Mirzaei et al., 2026), an effect particularly relevant given that SE is widely recognized as a stress-induced developmental response. These observations indicate that the physiological effects of GA biosynthesis inhibitors result from extensive reprogramming of hormone homeostasis and stress-related pathways rather than from reduced GA levels alone.

The role of GAs in the induction of SE varies not only among species but also among cultivars (Li et al., 2002; Fujimura and Komamine, 1975; Komai et al., 1996a; Tokuji and Kuriyama, 2003; Mitsuhashi et al., 2003) and even among individual plants within the same cultivar (Ishizaki et al., 2001; Milojević et al., 2011; Belić et al., 2020a, b). For instance, GA_3_ promoted SE in *Rosa chinensis* and *R. hybrida* (Li et al., 2002) as well as in spinach (Komai et al., 1996a), with clear cultivar-dependent responses. In carrot, GA_3_ inhibited SE in several cultivars but had a promotive effect in others (Fujimura and Komamine, 1975; Tokuji and Kuriyama, 2003; Mitsuhashi et al., 2003). Considerable variability was also observed among individual plants of the spinach cultivars ‘Nippon’ and ‘Matador’ in their response to GA_3_ (Ishizaki et al., 2001; Milić et al., 2017; Belić et al., 2020a, b). Even cell lines derived from individual seeds of spinach required different optimal concentrations of GA_3_ for the highest embryogenic response (Ishizaki et al., 2001; Belić et al., 2020b). A similar individual-dependent response was reported in *M. truncatula*, where GA_3_ was required for SE induction in line 2HA but inhibited embryo production in line M-19 (Nolan et al., 2014; Igielski and Kępczyńska, 2017). Because the GA biosynthesis inhibitor PAC also suppressed SE in line M-19, these contrasting responses most likely reflect differences in endogenous GA levels.

GAs also affect later stages of somatic embryo development, suppressing this process in many species, such as *Cedrela odorata* (Peña-Ramírez et al., 2011), *P. hortorum* (Hutchinson et al., 1997), carrot (Tokuji and Kuriyama, 2003), *Asparagus* spp (Li and Wolyn, 1997), and conifers (Pullman et al., 2005). In these species, GA biosynthesis inhibitors often enhanced embryo formation. In *P. hortorum*, GA_3_ inhibited somatic embryo formation, whereas PAC enhanced it when applied during the differentiation stage (Hutchinson et al., 1997). Similarly, GA_3_ arrested carrot somatic embryos at the globular stage, and this inhibition was reversed by treatment with uniconazole (Tokuji and Kuriyama, 2003).

As in zygotic embryogenesis, GAs frequently influence somatic embryo germination and conversion into plantlets (Eapen and George, 1994; Gaj, 2004; Corredoira et al., 2019; Guo et al., 2022; Ben Ali et al., 2023). For example, GA_3_ increased germination of cotyledonary-stage somatic embryos in *Quercus suber* and *Q. alba* (Corredoira et al., 2019; Ben Ali et al., 2023) and improved somatic embryo conversion in *Cinnamomum camphora* (Guo et al., 2024). This effect is largely associated with GA-induced activation of hydrolytic enzymes such as α-amylase, which enhances starch mobilization and supports embryo axis elongation and polarity establishment during plantlet regeneration (Kępczyńska and Zielińska, 2006).

Taken together, the available literature demonstrates that the effects of exogenous GA_3_ and GA biosynthesis inhibitors on SE are highly variable and largely dependent on endogenous GA levels, which differ markedly among species, cultivars, and genotypes. Given the significant role of endogenous GAs in SE, this aspect is discussed in greater detail in a separate section.

## 4 Interaction of GA with other factors in the promotion of somatic embryogenesis

GAs influence SE through complex interactions with numerous factors, including explant type, culture medium composition (particularly the type and concentration of PGRs), and external environmental conditions such as light and temperature. Leaves and petioles have been the most frequently used explants for GA_3_-mediated SE induction across a wide range of species (Rout et al., 1991; Hsia and Korban, 1996; Li et al., 2002; Ruduś et al., 2001). In spinach, however, roots exhibited the highest regeneration potential, particularly the apical segments of the lateral roots (Komai et al., 1996a; Xiao and Branchard, 1993).

### 4.1 Interaction of GA with other plant growth regulators

The interaction between GAs and other PGRs is a key factor in the regulation of SE. Because endogenous levels of GAs and other hormones vary among explants, the requirements for exogenous PGRs differ substantially (Jiménez, 2005). Approximately 80% of SE induction protocols rely on auxins, particularly 2,4-dichlorophenoxyacetic acid, either alone or combined with cytokinins (Gaj, 2004). GAs are not sufficient for SE induction; therefore, they are combined with auxins and/or cytokinins for SE induction and differentiation (Rout et al., 1991; Hunault and Maatar, 1995; Ruduś et al., 2001; Li et al., 2002). Optimal PGR combinations and concentrations are highly species- and cultivar-dependent. In *R. hybrida*, SE induction from petioles requires different GA_3_ and cytokinin combinations depending on the cultivar. For example, the highest regeneration frequency in cv. ‘Carefree Beauty’ was achieved with GA_3_ combined with either 2.3 µM thidiazuron (TDZ) or 2.2 µM 6-Benzylaminopurine (BA), whereas embryogenic callus formation in cv. ‘Red Sunblaze’ occurred only with GA_3_ + 54.5 µM TDZ (Hsia and Korban, 1996; Li et al., 2002). In contrast, cv. ‘Landora’ required the addition of naphthaleneacetic acid (NAA) to the GA_3_–BA combination to induce embryogenic callus (Rout et al., 1991). In spinach, optimal auxin–GA_3_ ratios varied by cultivar: high indole-3-acetic acid (IAA) (85.7 µM) + GA_3_ (10–100 µM) for cv. ‘Carpo’ (Xiao and Branchard, 1993), 30 µM NAA + 100 µM GA_3_ for cv. ‘Jiromaru’ (Komai et al., 1996b); and lower levels (20 µM NAA + 5 µM GA_3_) for cv. ‘Matador’ (Milojević et al., 2011, 2012; Belić et al., 2020a, b). Although interactions between GAs and ABA are less commonly reported, a strong synergistic effect between these hormones was demonstrated during SE induction from *M. truncatula* leaves, where the combination of NAA, BA, GA_3_, and ABA produced the highest number of somatic embryos (Nolan et al., 2014).

### 4.2 Impact of environmental cues on SE, GA biosynthesis and signaling

Plants respond to environmental cues such as light, temperature, abiotic and biotic stress by altering GA metabolism and signaling to synchronize their growth and development with environmental conditions (Shani et al., 2024). Although interactions between GAs and environmental factors also influence SE, these relationships remain relatively understudied. Light, perceived primarily via phytochromes, modulates GA metabolism and tissue sensitivity to GAs; red light typically promotes GA biosynthesis gene expression (e.g., during seed germination and plant deetiolation), while far-red light inhibits this process (Kamiya and Garcia-Martinez, 1999; Yamaguchi and Kamiya, 2000; Olszewski et al., 2002; Yang et al., 2018). Photoperiod affects endogenous GA levels, with long-day conditions (16 h of light) enhancing GA biosynthesis in long-day plants, such as spinach, promoting processes like stem elongation (**Figure 1**) (Wu et al., 1996; Lee and Zeevaart, 2002, 2005). Temperature also affects GA metabolism; at higher temperatures, GA biosynthesis and signaling generally increase to promote cell elongation, whereas at lower temperatures, GA levels are reduced, thereby slowing plant development (**Figure 1**) (Stavang et al., 2007; Izydorczyk et al., 2018; Wang et al., 2018; Lange et al., 2020). In addition, DELLA degradation can occur independently of GA/GID1, triggered by warmth, shade, light, and low nitrogen (Shani et al., 2024).

SE from root apices of spinach provides a clear example of the strong synergistic interaction between light and GAs (GA_3_ and GA_1_) in promoting SE. In the absence of both GAs and light, only root explants from the line with a high intrinsic capacity for SE regenerated somatic embryos, but at a frequency of merely 1% and 0.14 somatic embryos per explant (Belić et al., 2020b). However, in the presence of either light or GAs individually, explants from this line regenerated at a frequency of approximately 52%, producing 8.85 somatic embryos per explant. When both light and GAs were present, 100% of the explants responded, producing 40.73 somatic embryos per root explant (Belić et al., 2020b).

In most SE protocols, cultures are maintained at approximately 25 °C under fluorescent light with a 16-h light/8-h dark photoperiod, although optimal light intensity varies by species. Low light intensities (20–60 µmol m^-^² s^-^¹) favor SE induction in roses, whereas higher intensities (100–150 µmol m^-^² s^-^¹) are required in spinach, *Zea mays* and *M. sativa* (Ruduś et al., 2001; Joshi et al., 2010; Milojević et al., 2012). In spinach, regeneration of somatic embryos from root apices was significantly more efficient under long day conditions (16 h photoperiod) than under short day conditions (8 h photoperiod), typically commencing about four weeks earlier in explants of the same lines (Milojević et al., 2012). Moreover, cell lines at the individual plant level exhibited different preferences for light intensity: 100 µmol m^-^² s^-^¹ was generally optimal, while 150 µmol m^-^² s^-^¹ was supraoptimal in some lines but still within the optimal range in others (Milojević et al., 2012). Exceptions include continuous light with GAs promoting SE in *Iris germanica*, and dark conditions with GAs stimulating SE in *F. vulgare* by overcoming light inhibition (Shimizu et al., 1997; Hunault and Maatar, 1995).

## 5 Expression of genes involved in GA Metabolism and signaling during SE

Advances in gene sequencing have generated extensive literature on the expression of genes involved in GA metabolism and signaling during various stages of plant growth and development, significantly advancing our understanding of their functions (Locascio et al., 2013; Wang et al., 2015; He et al., 2022; Luo et al., 2023; Alabadí and Sun, 2025). Despite this progress, data on the expression of these genes during SE remain limited, and a clear picture of the role of GAs in this process has yet to be established.

The correlation between the acquisition of embryogenic competence and the expression of key genes encoding enzymes involved in GA metabolism has been examined in only a few studies. Most of these studies focused on the *GA oxidase* (*GAox*) genes—*GA20ox*, *GA3ox*, and *GA2ox*—as they encode the rate-limiting enzymes that control bioactive GA levels (Mitsuhashi et al., 2003; Wang et al., 2004; Nolan et al., 2014; Indoliya et al., 2016; Igielski and Kępczyńska, 2017; Belić et al., 2020b; Wu et al., 2023; Yue et al., 2023) (**Table 2**). Analyses of *GAox* gene expression in carrot and *M. truncatula* line M-19 revealed increased expression of *GA3ox* throughout all stages of somatic embryo development, while the expression levels of *GA20ox* and *GA2ox* remained unchanged. This suggests that enhanced GA biosynthesis acts as a stimulatory signal for SE induction in these species (Mitsuhashi et al., 2003; Igielski and Kępczyńska, 2017). Significant differences in *GA20ox* and *GA3ox* expression were also observed in embryogenic calli of two *Oryza sativa* subspecies (Indoliya et al., 2016) and in *Ormosia henryi* (Wu et al., 2023). As expected, increased expression of GA biosynthesis genes was detected in the *japonica* subspecies, which exhibits higher embryogenic potential compared with *indica* (Indoliya et al., 2016). In *O. henryi* embryogenic calli, expression of these genes initially decreased before gradually increasing during subsequent somatic embryo development (Wu et al., 2023). A strong correlation between increased GA biosynthesis and embryogenic competence was further supported by a recent study in *Agapanthus praecox*, where RNA interference–mediated silencing of *GA20ox* nearly abolished SE in RNAi lines compared with controls (Yue et al., 2023). Although exogenous GA_4_ partially restored somatic embryo numbers in RNAi lines, the response remained lower than in controls, highlighting the strong association between *GA20ox* expression and embryogenic competence in this species (Yue et al., 2023) (**Table 2**). Nonetheless, the precise mechanisms by which enhanced GA biosynthesis promotes SE remain unclear. In legumes, studies on zygotic embryogenesis have shown increased *GA3ox* mRNA accumulation in the suspensor of globular embryos, emphasizing the requirement for GAs during early zygotic embryo stages (Solfanelli et al., 2005; Henry and Goldberg, 2015). Similarly, reduced *GA20ox* and *GA3ox* expression was observed in an embryonic lethal rice mutant *osmpk6*, which impaired normal zygotic embryo development (Yi et al., 2016).

**Table 2:** Changes in GA metabolism gene expression and endogenous GA levels during SE in different plant species and the effects of exogenous GA_3_.

Species	Tissue or explant	Changes in GA metabolism gene expression during SE	Endogenous GA levels during SE	Relationship with embryogenic competence	Effect of exogenous GA_3_	Reference
*Daucus carota*	Embryogenic cultures	↑ *GA3ox*; *GA20ox* and *GA2ox* unchanged	Not investigated	Enhanced GA biosynthesis associated with SE induction	Not required for SE induction	Mitsuhashi et al., 2003
*Medicago truncatula*	Leaves of embryogenic M-19 line	↑ *GA3ox*; *GA20o*x and *GA2ox* unchanged	↑GA_53_, GA_19_, GA_1_, GA_3_, and GA_6_	High levels of endogenous GAs and increased expression of biosynthetic genes promotes SE	Inhibitory	Igielski and Kępczyńska, 2017
	Embryogenic callus of line 2H	↑ *GA2ox*	Not investigated	Increased GA catabolism associated with SE	Required	Nolan et al., 2014
*Oryza sativa*	Embryogenic calli	↑ *GA20ox* and *GA3ox*	Not investigated	GA biosynthesis promotes SE	Not required	Indoliya et al., 2016
*Ormosia henryi*	Embryogenic calli	Initial ↓ *GA20ox/GA3ox*, followed by gradual ↑	**↓**GA_1_, GA_3_, GA_4_, GA_7_, GA_9_, GA_15_, GA_19_, GA_20_, GA_24_, GA_53_	High levels of endogenous GAs and increased expression of biosynthetic genes promotes SE	Not required	Wu et al., 2023
*Agapanthus praecox*	Embryogenic callus of RNAi lines	RNAi-mediated silencing of *GA20ox* → strong ↓ SE	↓ GA_1_, GA_3_, GA_4_	The high expression of GA20ox is essential for SE	Partially restorative (GA_4_)	Yue et al., 2023
*Spinacia oleracea*	Root-derived cultures	↓ *GA20ox* and *GA3ox;* ↑ *GA2ox2*	↑GA_53_ GA_44_ GA_15_ GA_19_ GA_13_ GA_20_ GA_1_ GA_3_ GA_4_ GA_5_ GA_7_	Reduced GA biosynthesis and increased catabolism correlate with enhanced SE	Required for efficient SE induction	Belić et al., 2020b, 2022b
*Arabidopsis thaliana*	35S:AGL15 plants	↑ *AtGA2ox6*	↓ GA_1_, GA_4_, GA_8_, GA_9_, GA_19_, GA_20_, and GA_34_	Elevated GA catabolism associated with enhanced SE	Not required	Wang et al., 2004

Arrows indicate relative changes (↑ = increase; ↓ = decrease).

Notably, in the studies mentioned above, exogenous GA_3_ was either unnecessary (Mitsuhashi et al., 2003; Indoliya et al., 2016; Wu et al., 2023; Yue et al., 2023) or even inhibitory for SE induction, as reported for *M. truncatula* line M-19 (Igielski and Kępczyńska, 2017). In contrast, GAs are indispensable for SE induction from spinach roots. During SE induction from these explants, a consistent decrease in the expression of GA biosynthesis genes (*GA20ox* and *GA3ox*), coupled with increased expression of the catabolism gene *GA2ox2*, correlated positively with enhanced embryogenic potential (Belić et al., 2020b). Elevated expression of GA catabolism genes was also detected during SE in *M. truncatula* line 2H (Nolan et al., 2014) and *A. thaliana* (Wang et al., 2004). In *A. thaliana*, transgenic lines overexpressing *35S:AtGA2ox6* exhibited significantly higher embryogenic potential than *ga2ox* mutants (Wang et al., 2004; **Table 2**). These diverse and sometimes opposing expression patterns of key GA biosynthesis and catabolism genes across species (and even within species, as in *M. truncatula*) likely reflect differences in initial endogenous GA levels in explants. Thus, endogenous GA content appears to be a key regulator of the embryogenic response. In certain genotypes with high embryogenic potential, endogenous GA levels may suffice to support embryogenic callus proliferation and somatic embryo development, thereby eliminating the need for exogenous GA_3_ application. However, when exogenous GA_3_ is required for efficient SE induction (Nolan et al., 2014; Belić et al., 2020b), it profoundly influences GA metabolism gene expression. Decreased biosynthesis and increased catabolism gene expression can be attributed to feedback regulation mechanisms that maintain endogenous GA homeostasis in response to exogenous GA_3_. Comparable effects of exogenous GA_3_ on GA metabolism–related gene expression have been documented in different physiological processes, including growth and development of carrot roots (Wang et al., 2015), *Betula platyphylla* hypocotyls (Guo et al., 2015), *Eucalyptus grandis* shoots and roots (Liu et al., 2018), and rice leaves (He et al., 2022).

Expression of genes in GA signaling pathways, which encode the GID1 receptor and DELLA proteins, during SE remains underexplored, despite their central role in GA signal transduction (Locascio et al., 2013; Alabadí and Sun, 2025). In embryogenic calli of *Camellia sinensis*, *DELLA* gene expression was reduced relative to globular embryos (Awon et al., 2024). Similarly, in *O. henryi* embryogenic calli, *GID1* and *DELLA* gene expression patterns aligned with those of GA biosynthesis genes: upregulated *GA20ox* and *GA3ox* expression coincided with downregulated *DELLA* expression and upregulated *GID1* expression (Wu et al., 2023).

Collectively, these findings demonstrate diverse expression patterns of GA metabolism and signaling genes during SE, strongly modulated by endogenous and exogenous GA levels. As emphasized earlier, a critical factor in SE induction is the interaction of GAs with other PGRs (see section 4), and their combined effects on GA metabolism and signaling-related gene expression should not be overlooked. Auxins and GAs exhibit often synergistic interactions in regulating plant growth, development, and SE induction, with auxins often dominating by enhancing GA biosynthesis and signaling, particularly through upregulation of *GA20ox* and *GA3ox* and downregulation of *GA2ox* (Weiss and Ori, 2007; Ross et al., 2016; Hu et al., 2018b; He et al., 2022).

DELLA proteins serve as central integrators of these interactions by linking GA signaling to auxin pathways via interactions with AUXIN RESPONSE FACTOR (ARF) transcription factors (Oh et al., 2014; Hu et al., 2018), as well as with transcription factors involved in cytokinin, ABA, brassinosteroid, and ethylene signaling (Davière and Achard, 2016), thereby modulating GA metabolism gene expression. Specifically, DELLA interactions with ARF, PHYTOCHROME INTERACTING FACTOR (PIF4), or BRASSINAZOLE RESISTANT 1 (BZR1) stimulate GA biosynthesis gene expression (**Figure 1**) (Gallego-Bartolomé et al., 2012; Oh et al., 2014), whereas interactions with ABSCISICACID-INSENSITIVE (ABI) or ARABIDOPSIS RESPONSE REGULATOR (ARR) factors produce opposing effects (Tuan et al., 2018).

DELLA proteins can help maintain embryonic identity through ABA-dependent pathways. Consistent with their role in promoting seed dormancy and suppressing germination, DELLA proteins may stabilize the embryogenic state by reinforcing ABA responses and counteracting GA-mediated developmental transitions. This is supported by observations that high ABA-to-GA ratios are often associated with embryogenic competence (Nolan et al., 2014; Liu et al., 2018; Guo et al., 2022). Furthermore, DELLA proteins may act as molecular switches controlling the transition between embryogenesis and post-embryonic development. Increased GA biosynthesis promotes DELLA degradation and may activate germination-related programs, whereas DELLA accumulation may favor the maintenance of embryonic characteristics. Taken together, these observations suggest that DELLA proteins may act as integrators of hormonal and transcriptional networks controlling embryogenic competence, embryonic identity, and developmental transitions during SE. Future studies investigating DELLA expression, localization, and protein–protein interactions during SE will be essential to test these hypotheses.

Since GAs promote SE under light and long-day conditions in most studies reviewed here, the effect of light on GA metabolism gene expression must also be considered. Light promotes GA biosynthesis by upregulating *GA20ox* expression, which likely enhances endogenous GA levels (Wu et al., 1996; Lee and Zeevaart, 2005, 2007) and, in turn, facilitates SE induction.

In spinach root apices, light and GA_3_ significantly affected the expression levels of S*oGA20ox1*, *SoGA3ox1*, *SoGA2ox1*, *SoGA2ox2*, and *SoGA2ox3* (Belić et al., 2020b). In explants cultivated on PGR-free medium, the expression levels of all genes were significantly higher under long-day photoperiod (16 h light/8 h dark) than in darkness (Belić et al., 2020b). However, under a long-day photoperiod, the expression of both *SoGA20ox1* and *SoGA3ox1* significantly decreased, while the expression of *SoGA2ox2* and *SoGA2ox3* significantly increased in explants cultivated on medium for SE induction (20 μM NAA + 5 μM GA^3^) compared with those cultivated on PGR-free medium (Belić et al., 2020b).

Considering the extensive evidence for the effects of GAs, other phytohormones, and light on the expression of genes involved in GA metabolism and signaling, future research should focus on integrative gene expression analyses to better elucidate the nature of these interactions and their regulatory roles in SE.

## 6 Transcription factors

A complex network of interactions among transcription factors such as LEAFY COTYLEDON (LEC), BABY BOOM (BBM), FUSCA3 (FUS3), AGAMOUS-LIKE15 (AGL15), and ABI3 collectively induces SE and embryo development (Stone et al., 2001; Boutilier et al., 2002; Guo et al., 2013; Radoeva and Weijers, 2014; Horstman et al., 2017a, b; Gulzar et al., 2020; Salaün et al., 2021).

BBM plays a central regulatory role by establishing somatic cell totipotency and controlling the expression of other embryogenesis-related genes (Horstman et al., 2017b). LEC1/L1L, ABI3, FUS3, and LEC2, collectively referred to as LAFL proteins, coordinately regulate embryogenesis. Expression of *BBM, LEC1*, or *LEC2* alone is sufficient to induce somatic embryogenesis, whereas AGL15, FUS3, and ABI3 primarily act as enhancers of this process (Horstman et al., 2017a). LEC promotes an embryonic developmental program on somatic cells (Gaj et al., 2005; Stone et al., 2008; Wójcikowska and Gaj, 2015), whereas FUS3 and ABI3 are involved in the conversion of somatic embryos into seedlings. In vegetative tissues, the activity of these transcription factors is suppressed by chromatin-remodeling enzymes, primarily through the action of PICKLE (PKL) (Rider et al., 2003; Henderson et al., 2004).

Most transcription factors active during SE interact extensively with auxin metabolism and signaling pathways (Gliwicka et al., 2013; Xu et al., 2019a). However, several studies have demonstrated their interaction with GAs, with LEC1 playing a central role as a key regulator of embryo and seed development (Jo et al., 2019, 2020). LEC1 initiates the expression of other seed development regulators, including ABI3, FUS3, LEAFY COTYLEDON LIKE1 (LIL1), and LEC2 (Pelletier et al., 2017). LEC1 directly interacts with DELLA proteins, which inhibit its activity, whereas bioactive GAs that accumulate during early embryo development release LEC1 from this inhibition (Hu et al., 2018a). Overexpression of *LEC1* leads to reduced endogenous ABA levels and increased levels of GAs, IAA, and cytokinins (Xu et al., 2019b). FUS3 also integrates hormonal signals: its expression is induced by ABA and repressed by GAs (Chiu et al., 2016). Overexpression of *CsFUS3* in *Citrus* spp. creates a favorable environment for somatic embryo regeneration, at least in part by establishing a high ABA-to-GA ratio (Liu et al., 2018). The FUS3 protein physically interacts with two RY elements (CATGCATG) present in the promoter of the *AtGA3ox2* gene, thereby repressing its expression; this repression is essential for normal embryo development in *A. thaliana* (Curaba et al., 2004).

Treatment of soybean cotyledon explants with GA_3_ significantly reduced the expression of *AGL15*, *AGL18*, *ABI3*, and *FUS3*, whereas treatment with PAC increased the expression of these genes and suppressed the expression of *GA3ox2* (Zheng and Perry, 2014; Zheng et al., 2016). AGL15 influences ethylene biosynthesis and perception (Zheng et al., 2013), and ethylene and GAs most often act antagonistically during SE (Weiss and Ori, 2007). AGL15 is thought to promote SE by stimulating the expression of A*tGA2ox6* and thereby reducing bioactive GA levels (Wang et al., 2004). Similarly, AGL18 reduces GA levels during SE by repressing *GA3ox2* expression; homozygous *ga3ox2* knockout lines of *A. thaliana* produced significantly more somatic embryos than wild-type plants (Paul et al., 2022). Although AGL18 does not directly regulate *GA2ox6*, it interacts with AGL15 to upregulate its transcription (Paul et al., 2022). BBM promotes SE, at least in part, through modulation of the GA signaling pathway. In soybean hairy roots overexpressing *GmBBM7*, endogenous GA levels were significantly lower than in wild-type controls (Zhou et al., 2023). Furthermore, the GA signaling repressor RGA-LIKE3 (RGL3) interacts with ABI3, and BBM regulates *ABI3* expression, suggesting an indirect role for BBM in modulating GA levels during SE (Hu et al., 2021).

PKL also exerts an inhibitory effect on SE through regulation of GA signaling (Ogas et al., 1999; Henderson et al., 2004). PKL-mediated repression of *LEC1* is associated with H3K27 trimethylation, whereas VAL1 and VAL2 interact with histone deacetylases HDA19 and HDA6 (Ogas et al., 1999). Roots of *pkl* mutants spontaneously form callus and somatic embryos (Thomas and Jiménez, 2005), and *val1/val2* double mutants regenerate somatic embryos from the shoot apical meristem (Suzuki et al., 2007). Mutant *pkl* seedlings display numerous embryonic characteristics, including storage compound accumulation (Ogas et al., 1999). DELLA proteins interact with PKL to inhibit its activity, and GAs relieve this repression (Zhang et al., 2014). GA biosynthesis inhibitors enhance SE in *pkl* and *val1/val2* mutants (Ogas et al., 1997; Suzuki et al., 2007). A low ABA-to-GA ratio inhibited PKL expression while stimulating *GA2ox* expression, which correlated with SE induction in *M. truncatula* (Nolan et al., 2014).

## 7 Endogenous GAs

Endogenous hormones regulate gene activity, thereby influencing a range of metabolic processes and ultimately affecting the induction, maintenance, and expression of embryogenic potential in plant cells (Jiménez, 2005). Since the role of GAs in the induction of SE can be either promotive or inhibitory depending on the plant species, different cellular requirements for endogenous GA levels have been observed during the acquisition of embryogenic competence.

A correlation between increased levels of endogenous GAs and the induction of SE has been demonstrated in several studies (Jiménez and Bangerth, 2001; Jiménez et al., 2005; Igielski and Kępczyńska, 2017; Kępczyńska and Orłowska, 2021; Belić et al., 2022b; Awon et al., 2024). In leaves of embryogenic and non-embryogenic lines of *M. truncatula*, all GA metabolites (precursors, bioactive, and inactive) from both the 13-non-hydroxylated and 13-hydroxylated GA pathways were detected during a three-week induction period, with clear differences in the levels of individual metabolites between the two lines (Igielski and Kępczyńska, 2017; Kępczyńska and Orłowska, 2021). Seven days after the onset of induction period, the embryogenic line showed a significant increase in the precursors GA_53_ and GA_19_, as well as in bioactive GAs (GA_1_, GA_3_, and GA_6_), relative to the non-embryogenic line (Igielski and Kępczyńska, 2017; Kępczyńska and Orłowska, 2021). Similarly, elevated endogenous GA levels were detected in embryogenic calli of maize (Jiménez and Bangerth, 2001), *Citrus sinensis* (Awon et al., 2024), and spinach (Belić et al., 2022b). The predominant bioactive GA in embryogenic explants of spinach and *M. truncatula* was GA_3_, suggesting that its increased levels are critical for the acquisition of embryogenic competence (Kępczyńska and Orłowska, 2021; Belić et al., 2022b).

The precise mechanisms activated by increased endogenous GA levels during SE induction have yet to be fully clarified. One potential mechanism triggered by higher endogenous GA levels in embryogenic explants is negative feedback regulation, which inhibits the expression of GA biosynthesis genes and thereby maintains the GA homeostasis necessary for SE induction. Another mechanism involves the ability of endogenous GAs to influence the levels and activity of other phytohormones, particularly auxins. As previously noted, auxins strongly affect GA metabolism, whereas GAs exert a lesser influence on auxin biosynthesis but can modulate auxin levels and spatial distribution through regulation of polar auxin transport (Willige et al., 2011; Löfke et al., 2013; Mäkilä et al., 2023), thereby establishing feedback regulation between these two hormones (Hu et al., 2018b). Willige et al. (2011) demonstrated that a gene required for directing PIN-FORMED (PIN) proteins to vacuoles is regulated by DELLA proteins, which in turn modulates the asymmetric expression pattern of PIN proteins in response to endogenous GA levels. Through this mechanism, GAs can influence the formation of auxin gradients during SE induction, which is crucial for establishing bilateral symmetry in embryogenic cells (Liu et al., 1993; Fischer and Neuhaus, 1996).

A further mechanism involves interactions between GAs and transcription factors active during SE, with LEC1 playing a central role. During seed development, elevated endogenous GA levels induce *LEC1* gene expression, which positively regulates multiple aspects of embryo and endosperm development (Hu et al., 2018a). In the cotyledonary stage of zygotic embryos in *A. thaliana*, increases in GA_1_, GA_3_, and GA_4_ concentrations correlate with enhanced LEC1 activity. The effect of elevated bioactive GA levels is manifested through destabilization of DELLA proteins, which interact with LEC1 and inhibit its activity. In this way, GAs relieve repression of LEC1, thereby enabling the induction of genes involved in auxin biosynthesis (e.g., *YUCCA*), which are essential for normal embryo development (Hu et al., 2018a). Although these mechanisms have been extensively investigated in other physiological processes, studies that directly link them with SE are still lacking.

In contrast to the examples above, decreases in GA levels and increases in GA catabolism have been correlated with SE induction in *Pimpinella anisum*, *D. carota* (Noma et al., 1982), and *O. henryi* (Wu et al., 2023). Similarly, in embryogenic calli of *Phoenix dactylifera*, GA_3_ levels were eight-fold lower than in non-embryogenic calli (Zein El Din et al., 2023). Nevertheless, increases in GA_3_ were detected in somatic embryos of *P. dactylifera* (Zein El Din et al., 2023) and *C. camphora* (Guo et al., 2022). Such elevated levels potentially correlate with the requirement for GAs during early embryo development stages as well as during subsequent germination and seedling growth.

By activating various mechanisms, GAs can play diverse roles depending on the plant developmental phase and the specific physiological process. For example, in hybrid poplar perennial shoots, GA_3_ and GA_6_ accumulate during apical bud formation and upregulate *GA2ox* genes. In turn, GA2ox inactivates GA_1_ and GA_4_, ultimately inhibiting shoot branching. Apical bud decapitation reduces GA_3_ and GA_6_ levels, suppresses *GA2ox* expression, elevates GA_1_ and GA_4_, and thereby promotes branching (Katyayini et al., 2020). Similarly, in carrot, direct SE from explants is stimulated by low endogenous GA levels (Tokuji and Kuriyama, 2003), whereas indirect SE induction is favored by elevated GA levels (Mitsuhashi et al., 2003).

The variable effects of endogenous GA on SE may be explained, at least in part, by the fact that embryogenic competence is determined not only by endogenous GA levels but also by their balance with other phytohormones within the target tissue. Similar to their well-established role in seed biology, relatively high ABA-to-GA ratios appear to favor the acquisition and maintenance of embryonic identity, whereas lower ABA-to-GA ratios and increased GA signaling are generally associated with developmental programs related to embryo germination and post-embryonic growth. Such relationships have been reported in species including *A.thaliana* (Braybrook and Harada, 2008), *Citrus* spp (Liu et al., 2018) and *Cinnamomum camphora* (Guo et al., 2022). However, this pattern is not universal. For example, low ABA-to-GA ratios were also associated with enhanced somatic embryogenesis in *M. truncatula* (Nolan et al., 2014) and *Ormosia henryi* (Wu et al., 2023), indicating that the hormonal requirements for embryogenic competence differ among species.

**Figure 2:**
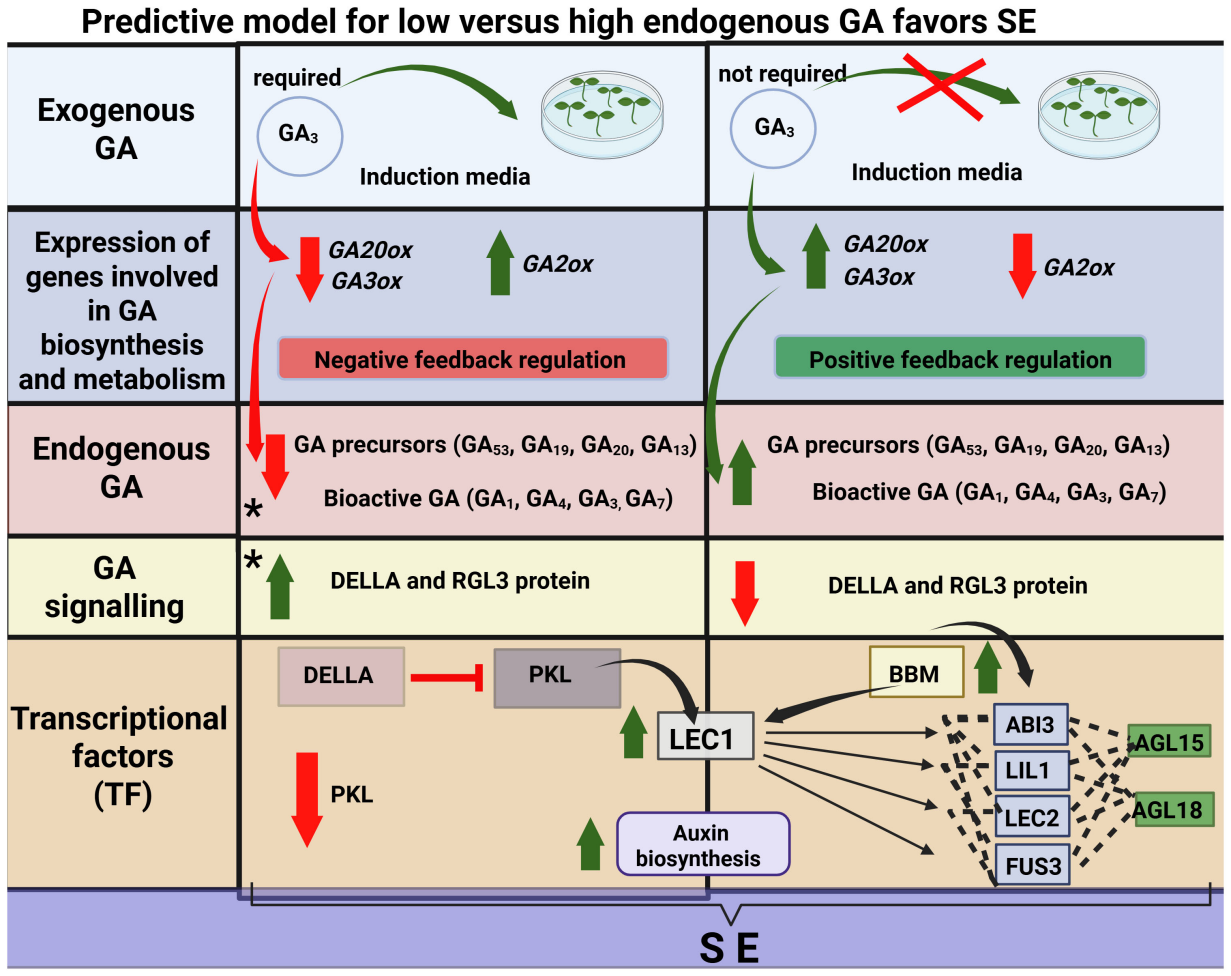
Proposed conceptual model explaining the contrasting roles of endogenous gibberellins (GA) during somatic embryogenesis (SE). SE may be promoted through two distinct regulatory scenarios associated with different (low or high) GA levels. In the first scenario, exogenous GA_3_ is required for SE induction. Exogenous GA_3_ activates a negative feedback mechanism that suppresses the expression of GA biosynthesis genes, resulting in reduced endogenous GA levels. Low endogenous GA levels favor DELLA accumulation, leading to the repression of developmental programs associated with germination and post-embryonic growth through PKL inhibition, while promoting the acquisition and maintenance of embryogenic competence. In the second scenario, exogenous GAs are not required for SE induction. Their absence activates a positive feedback mechanism that enhances the expression of GA biosynthesis genes, leading to increased endogenous GA levels and DELLA degradation. The release of DELLA-mediated repression allows the activation of embryogenesis-related transcription factors, including LEC1, LEC2, BBM, AGL15, and AGL18. In particular, LEC1 promotes the expression of genes involved in auxin biosynthesis, thereby stimulating embryo development. Green arrows indicate positive regulation, whereas red arrows indicate negative regulation. The model represents a working hypothesis derived from currently available literature and requires further validation through studies integrating hormone profiling, GA signaling analyses, gene expression data, and diverse genetic backgrounds. ^*^ Endogenous bioactive GA levels have not been directly measured. Reduced GA levels and DELLA accumulation are inferred from gene-expression data and require experimental validation. The graph was created in BioRender (Agreement number: TE29VPURAM) https://BioRender.com/wp6sntz; last accessed (25.06.2026.).

Based on the available literature, we propose a conceptual model in which SE can be promoted through two distinct regulatory scenarios associated with different (low or high) GA levels (**Figure 2**). In the first scenario, exogenous GA_3_ is required for SE induction. The presence of GA_3_ in the induction medium activates a negative feedback mechanism that suppresses the expression of genes involved in GA biosynthesis, resulting in reduced endogenous GA levels (Nolan et al., 2014). Low endogenous GA levels favor DELLA accumulation, leading to the repression of developmental programs associated with germination and post-embryonic growth through PKL inhibition, while promoting the acquisition and maintenance of embryogenic competence (Ogas et al., 1999; Henderson et al., 2004; Zhang et al., 2014). Under these conditions, DELLA proteins may contribute to the establishment of an embryogenic state by integrating hormonal signals and reinforcing regulatory pathways associated with embryo development (**Figure 2**). It should be noted that this scenario is based primarily on gene-expression data, whereas direct measurements of endogenous bioactive GA levels in systems requiring exogenous GA_3_ for SE induction remain very limited. Consequently, the proposed reduction in endogenous GA levels and the associated DELLA accumulation should be regarded as a hypothesis that requires validation through integrated analyses of endogenous GA content, GA metabolism, and GA signaling.

In the second scenario, exogenous GAs are not required for SE induction (Igielski and Kępczyńska, 2017; Yue et al., 2023; Awon et al., 2024). Their absence triggers a positive feedback mechanism that enhances the expression of genes involved in GA biosynthesis, resulting in increased endogenous GA levels and subsequent DELLA degradation. The removal of DELLA-mediated repression may release key embryogenesis-related transcription factors, including LEC1, LEC2, BBM, AGL15, and AGL18, thereby promoting the embryogenic developmental program (Jo et al., 2019, 2020). In particular, LEC1 has been shown to induce the expression of genes involved in auxin biosynthesis, a process essential for normal embryo development (Hu et al., 2018a). Thus, elevated endogenous GA levels may favor SE induction by activating key embryogenesis-related transcription factors and their downstream regulatory networks (**Figure 2**).

These contrasting responses suggest that the effect of GA on SE cannot be explained solely by their absolute concentration, but rather by their integration within species-specific regulatory networks involving hormonal crosstalk, transcriptional regulation, and developmental context. Indeed, endogenous hormone levels alone cannot fully explain the acquisition of embryogenic competence. The same embryogenesis-related genes may be regulated by different hormonal pathways in different species. For example, auxin induces WUSCHEL expression during SE in *Arabidopsis* (Su et al., 2009; Tang et al., 2020), whereas cytokinins perform this role in *M. truncatula* (Chen et al., 2009; Rose, 2019). Similarly, downregulation of *PKL* expression is associated with high ABA-to-GA ratios in *Arabidopsis* but with low ABA-to-GA ratios in *M. truncatula.* These observations indicate that species-specific gene regulatory networks determine how hormonal signals are integrated to control embryogenic competence and somatic embryo development.

Although the proposed model provides important insights into how contrasting endogenous GA levels may stimulate SE, it should be considered a conceptual hypothesis rather than a universally applicable model. The considerable diversity of embryogenic responses observed among plant species suggests that additional regulatory mechanisms and species-specific genetic networks are also involved in determining embryogenic competence. Moreover, relatively few studies have simultaneously examined endogenous GA levels, the expression of GA metabolism genes, and components of the GA signaling pathway during SE. Future studies integrating hormone profiling, GA signaling analyses, transcriptomic approaches, and diverse genetic backgrounds will be necessary to validate and refine this model and to determine its applicability across plant species.

## 8 Conclusion

SE is a complex developmental process whose regulation relies on the precise interplay of phytohormones, with GAs playing a significant, but sometimes contradictory role. Available evidence indicates that exogenous application of GAs can exert either stimulatory or inhibitory effect on the induction and development of somatic embryos, depending on the plant species, genotype, and developmental stage. These divergent responses highlight the need to consider exogenous GAs in the context of endogenous GA homeostasis, which contributes substantially to genetic variation in regeneration efficiency.

GAs influence SE through crosstalk with other phytohormones and by modulating various transcription factors during this process. Although the mechanisms of GA action have been extensively characterized in other developmental processes, their role in SE remains poorly understood. Further research, employing an integrated approach that combines hormonal, molecular, and developmental analyses, is essential to elucidate the function of GAs in SE and thereby improve protocols for plant regeneration.
